# Comparison of Epidemiological Variations in COVID-19 Patients Inside and Outside of China—A Meta-Analysis

**DOI:** 10.3389/fpubh.2020.00193

**Published:** 2020-05-08

**Authors:** Ali Ahmed, Areeba Ali, Sana Hasan

**Affiliations:** ^1^Department of Operations & Information Systems, Manning School of Business, University of Massachusetts Lowell, Lowell, MA, United States; ^2^Institution of Industrial Biotechnology (IIB), Government College University Lahore, Lahore, Pakistan; ^3^University College of Medicine and Dentistry, The University of Lahore, Lahore, Pakistan

**Keywords:** COVID-19, 2019-nCoV, epidemiological characteristics, symptoms, meta-analysis

## Abstract

The objective of this study is to compare the epidemiological variations in COVID-19 patients reported in studies from inside and outside of China. We selected COVID-19 observational studies from eight countries, including, China, Italy, Australia, Canada, Korea, Taiwan, Singapore, and the USA, comprising a total of 13 studies and performed a meta-analysis for age, gender, fatality rate, and clinical symptoms of fever, cough, shortness of breath, and diarrhea. The meta-analysis shows that there are differences in symptoms and other characteristics reported by the patients of COVID-19 inside and outside China. Patients in China have a higher proportion of fever, cough, and shortness of breath as compared to patients outside of China. However, we found the opposite results for the gastrointestinal symptoms such as Diarrhea. Patients outside of China have a significantly higher proportion of Diarrhea as compared to patients within China. We also observed gender disparity among our studies, with the male population being more susceptible than the female population. Moreover, the analysis suggests that the fatality rate in China is relatively lower as compared to the fatality rate in other countries. These findings also suggest that the clinical symptoms of COVID-19 should not be generalized to fever, shortness of breath, and cough only but other symptoms such as diarrhea are also prevalent in patients with COVID-19.

## Introduction

The 2019 outbreak of the novel COVID-19 pandemic created challenges for the scientific community and the healthcare professionals (1). In the beginning of December 2019, the novel Coronavirus COVID-19 was first reported in China's Wuhan City (2, 3). There were reports of clusters of cases with unknown kinds of pneumonia with affiliation to the Huanan Animal wholesale food market (4). Within few days, the Chinese Health Authority announced this disease to be caused by a novel Corona Virus named 2019-nCoV (5) which has 70% similarity to the human-derived Severe Acute Respiratory Syndrome like coronavirus (SARS-CoV) and 88% similarity to the bat derived SARS-CoV (4, 6). As of March 22nd, 2020, the virus has spread to more than 187 countries and the total number of infected people are 294,110. The number of deaths caused by the virus as of March 22, 2020, is 12,944 (5).

The initial research conducted on the novel-COVID19 virus was aimed to better understand the epidemiological characteristics of the affected population (3, 7–10). The scarcity of first-hand patient's dataset, limited information, and the ever-evolving situation created hurdles in analyzing COVID-19 patient's health characteristics. Moreover, some of the initial observational studies on the epidemiological characteristics of the patients reported mixed results (10). For instance, Sun et al. (11) found that gastrointestinal symptoms, such as diarrhea, are common among patients with COVID-19. However, early studies conducted by researchers in China didn't include digestive problems as the major health symptoms for COVID-19 (2, 9). Therefore, in this study, we have conducted a meta-analysis of the observational research studies which were published in the first 3 months after the outbreak of COVID-19. We have analyzed the studies which have documented the epidemiological characteristics of patients infected by the COVID-19 virus, such as the patient's age, gender, major symptoms, and the fatality rate. The previously conducted meta-analysis have been geographically restricted to China and used a relatively smaller data set. According to the best of our knowledge, this meta-analysis is the first study that analyzes heterogeneity in COVID-19 patient's characteristics by comparing studies conducted in China and seven other countries. Therefore, this meta-analysis presents a timely synthesis of currently available observational studies on COVID-19.

At the time of writing this article, March 2020, there were already three meta-analyses related to the clinical symptoms of COVID-19. Yang et al. (12) showed the prevalence of comorbidities in COVID-19 infected patients based on eight studies from China. They also assessed that comorbidities such as hypertension, diabetes, cardiovascular diseases, and respiratory system diseases are a risk factor for severe patients as compared to non-severe patients. Rodriguez-Morales et al. (10) included 19 studies in his meta-analysis. They compared the clinical symptoms of COVID-19 including fever, cough, sore throat, myalgia, headache, diarrhea, and dyspnea. They also assessed the prevalence of comorbidities in COVID-19 confirmed cases. They included 18 studies from China and only 1 from Australia. While Li et al. (13) did a meta-analysis based on 10 studies from Chinse hospitals. They also compared the clinical characteristics of COVID-19 patients including their age, fatality rate, and discharge rate. Therefore, our meta-analysis is different because we are presenting a comparison of the epidemiological characteristics of patients inside and outside of China.

Our work is similar and close to the work of Badawi and Ryoo (14), they performed a similar type of meta-analysis for MERS-CoV (Middle Eastern Respiratory Syndrome-Coronavirus). In their study, they analyzed the symptoms of patients and compared the prevalence of comorbidities in MERS-CoV studies from different countries. His findings were that chronic diseases such as cardiac diseases, obesity, hypertension, and diabetes had a high prevalence in MERS-CoV patients (14).

Our meta-analysis of 13 studies not only confirms the findings of the previous meta-analysis on COVID-19 but also brings new insight for healthcare professionals and researchers. Our findings confirm that there are more men reported to be infected by the virus as compared to women and the age range for most patients is between 45 and 60 years regardless of inside or outside China. We also confirm the findings of the previous meta-analyses that most patients had one or more reported comorbidities. However, the three most commonly reported symptoms, fever, cough, and shortness of breath are not reported in a similar proportion inside and outside China. Fever was much less likely reported in the patients outside China. We found similar results for Cough and Shortness of Breath. We also found that Diarrhea was a commonly reported symptom in patients outside China.

The rest of the paper is organized in the following sections; in the next section, we have detailed our article selection methodology and search strategy. In section Detailed Analysis of Each Included Study, we have described each of the selected studies in detail. In Section Results, we presented the results by developing Forest Plot to compare studies inside and outside China. In the last section, we have presented a discussion on our results and concluded the meta-analysis.

## Data and Methods

### Information Sources and Search Strategy

**Figure 1:**
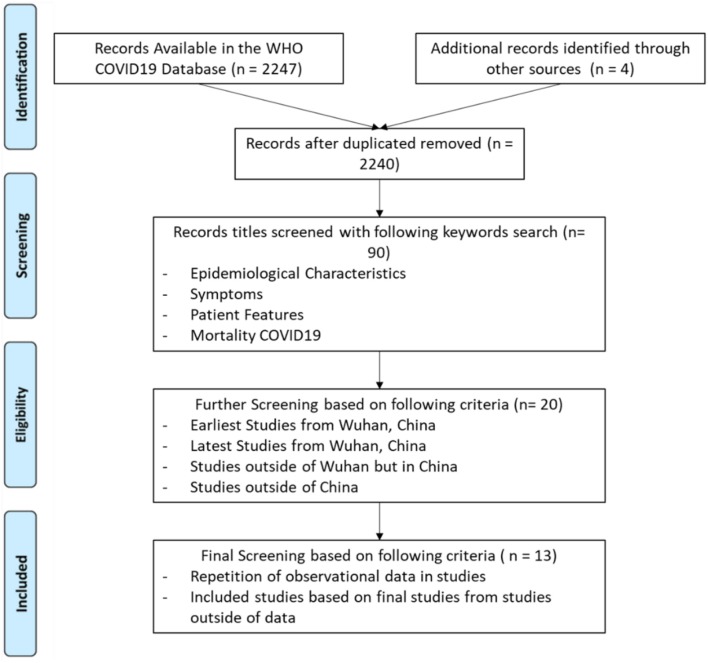
Systematic literature review process the flow sheet diagram represents the systematic review of literature for the characteristics of clinical symptoms (16).

To make our search comprehensive, we started our search from the World Health Organization database, which has combined research papers on the COVID-19, SARS, MERS, and related diseases (15). There were a total of 2,247 papers that were on the topic of coronavirus COVID-19 till March 20th, 2020. We also searched for observational studies on Google Scholar, PubMed, and ScienceDirect on COVID-19 which were published after December 2019. After combining the papers from both sources, we identified a total of 2,251 research studies. We selected only those studies, which are observational and discussed epidemiological characteristics of patients, we also use the keyword search for “symptoms,” “patient features,” “mortality,” and “epidemiological characteristics.” These keywords filtering shortlisted our database to 90 articles. In these articles, most of the papers obtained the data from Wuhan China. We further shortlisted our search by selecting: the earliest data of COVID-19 in Wuhan, the latest data of Wuhan, data of different provinces of China, and COVID-19 data from different countries of the world. This brought down our data to 20 papers. During our search, we also encountered studies which were only conducted on the same patients, or specific types of patients, such as pregnant women, patients with gastrointestinal problems, or children having COVID-19. Since we are looking for studies with the general population so, these studies do not fit in our selection criteria and they may make our results biased. Therefore, our final selection of studies included 6 studies conducted in China and 7 studies from other countries of the world. The flow chart of the selection process is shown in Figure 1.

### Data Extraction and Study Analysis

Data extraction and the evaluation of the quality of research was conducted independently by two investigators (Areeba Ali and Ali Ahmed). Microsoft Excel was used to store all the available information, including the total number of patients, gender percentage, median age, clinical symptoms, and fatality rate from each study. Any disagreement on inclusion or exclusion of the study was resolved by consulting with another investigator (Sana Hasan). The final sample included COVID-19 observational studies from China, Taiwan, Singapore, Canada, USA, Australia, Italy, and South Korea, as shown in Table 1.

**Table 1:** Studies Included in the meta-analysis.

**Study ID**	**References**	**Date of publication**	**City, Country**	**Number of patients**
**STUDIES INSIDE CHINA**				
Study 1	Huang et al. (3)	1/24/2020	Wuhan, China	41
Study 2	Li et al. (9)	1/29/2020	Wuhan China	425
Study 3	Wang et al. (17)	2/7/2020	Wuhan, China	138
Study 4	Chen et al. (18)	2/15/2020	Wuhan, China	99
Study 5	Guan et al. (2)	2/20/2020	Mainland China	1,099
Study 6	Wu et al. (19)	2/29/2020	Jiangsu, China	80
**Sub-total**				**1,882**
**STUDIES OUTSIDE CHINA**				
Study 1	Kong et al. (20)	2/21/2020	South Korea	28
Study 2	Young et al. (21)	3/3/2020	Singapore	18
Study 3	COVID-19 National Incident Room Surveillance (22)	3/5/2020	Australia	71
Study 4	Lin et al. (23)	3/6/2020	Toronto, Canada	135
Study 5	Su and Lai (24)	3/14/2020	Taiwan	10
Study 6	Livingston and Bucher (25)	3/17/2020	Lombardy, Italy	22,512
Study 7	Arentz et al. (26)	3/19/2020	Washington, USA	21
**Sub-total**				**22,795**

## Detailed Analysis of Each Included Study

The data collected from all the 13 studies which include age range, median age, sex ratio, and fatality rate are shown in Table 2, while the detailed clinical symptoms are shown in Appendix—Table A1. In the following sub-sections, we have provided a detailed summary of each of the studies included in our paper.

**Table 2:** Age, gender and fatality rate.

**References**	**Age range**	**Median age**	**Sex male (%)**	**Fatality rate(%)**
Huang et al. (3)	41–58	49	30 (73%)	6 (15%)
Li et al. (9)	26–82	56	281 (66%)	–
Wang et al. (17)	42–68	56	75 (54%)	6 (4%)
Chen et al. (18)	21–82	56	67 (68%)	11 (11%)
Guan et al. (2)	35–58	47	637 (58%)	15 (1%)
Kong et al. (20)	20–73	42.6	15 (54%)	0 (0%)
Wu et al. (19)	30–62	46.1	38 (48%)	0 (0%)
Young et al. (21)	31–73	47	9 (50%)	0 (0%)
COVID-19 National Incident Room Surveillance (22)	0–94	45	–	2 (3%)
Lin et al. (23)	23–49	28	59 (44%)	0 (0%)
Su and Lai (24)	–	49	3 (30%)	0 (0%)
Livingston and Bucher (25)	0–90	50	13,282 (59%)	1,625(7.2%)
Arentz et al. (26)	43–92	70	11 (52%)	11 (52%)
*Average* ± *SE*.	–	47 ± 7	–	0.07 ± 0.14

### Studies From China

Study 1 was conducted by Huang et al. (3), reported 41 laboratory-confirmed COVID-19 infection cases. The dataset used in the study was from the patients who were admitted to the hospital between December 16th, 2019 to January 2nd, 2020 in Wuhan, Hubei province, China. They found some patients had underlying diseases, diabetes, cardiovascular disease, and hypertension. They also reported that all 41 patients had exposure to the Huanan seafood market, where this virus was originated first. Common symptoms reported by the authors were fever (98%), cough (76%), and fatigue (44%). The less common symptoms were sputum production (28%), headache (28%), hemoptysis (5%), and diarrhea (3%). It also included Radiological reports and blood reports from the patients and comparison of reports of ICU and Non-ICU.

Study 2 conducted by Li et al. (9), included the first 425 confirmed cases till January 22nd, 2020 in the Wuhan, Hubei province, China. The study analyzed the key epidemiological data and described the characteristics of all the cases. They estimated the doubling time of epidemic and the basic reproductive number. The median age in the dataset was 59 years and there were 56% male patients in the dataset. It also showed that 55% of patients had a link to the Huanan seafood market. The report shows that the incubation period was 5.2 days and the epidemic doubled in size every 7.4 days. They show the spread of diseases through human to human transmission, and also stressed the need for interventions to reduce the transmission.

Study 3 conducted by Wang et al. (17) included 138 confirmed patients at Zhongnam Hospital of Wuhan University in Wuhan China from January 1 to January 28, 2020. Wang et al. (17), analyzed the demographical, epidemiological, clinical, laboratory, radiological, and treatment data. The results of critical and non-critical patients were compared. The report showed the median age was 56 years with the range being 42–68 years old. It reported three common symptoms like fever, fatigue, and dry cough. The mortality rate reported was 4.3%.

Study 4 by Chen et al. (18), reported 99 cases from Wuhan Jinyintan Hospital from January 1st to January 20th, 2020. They reported that 49% of patients had exposure to the Huanan seafood market. Fifty-one percentage of patients were reported to have chronic diseases. The most common symptoms reported were fever, cough, shortness of breath, myalgia, confusion, headache, sore throat, nasal congestion, diarrhea, and nausea or vomiting. The report showed an 11% fatality rate.

Study 5 by Guan et al. (2), analyzed data of 1,099 laboratory-confirmed cases from 552 hospitals in 30 provinces through January 29, 2020. They reported the median age of patients was 47 years and 58.1% were reported to be male patients. The most common symptoms reported were fever in 88.7% patients, cough in 67.8%, and diarrhea in 3.8% patients. The research also concluded that there were not many abnormal radiological findings in patients. The fatality rate of patients was 1.4%.

Study 6 conducted by Wu et al. (19), included the 80 confirmed cases of Jiangsu province in China from January 22nd to February 14th, 2020. The median age of patients was 46 years. The research shows that 47.5% of cases had chronic diseases. The main symptoms of patients included fever in 78.75%, cough in 63.75%, shortness of breath in 37.50%, 22.50% had myalgia and 16.25% had a headache. The research showed the abnormalities in radiological reports was 68.75% and there were no deaths.

### Studies Outside China

#### A Study From South Korea

Kong et al. (20) study reported the statistics till February 14th, 2020 that 28 confirmed patients are reported from the Korea Center of Disease and Control. The study mentions the sex, age-range, and common symptoms of all the patients along with the identification of route of transmission and incubation period. The research shows that there were 53.6% of men with a range of patients in age 20–73 years old. Kong et al. (20), also reported that 35.7% of patients had chronic diseases. The most common symptoms reported were fever, headache, cough, sore throat, sputum production, fatigue, and chills.

#### A Study From Singapore

Young et al. (21) reported 18 confirmed cases of COVID-19 infection. The patients were diagnosed using real-time RT-PCR at 4 hospitals in Singapore from January 23rd to February 3rd, 2020 and the final follow up was on February 25th, 2020. The study analyzed the clinical, laboratory, and radiological data. It has summarized the use of supplemental oxygen, ICU, and the use of empirical treatment with lopinavir-ritonavir. The research shows the median age to be 47 years with 50% male patients. The study also shows that the virus was detected in the stool of 50% patients and the blood of 8% patients by PCR but not in urine. The 4% of patients treated with lopinavir-ritonavir developed abnormal functioning and nausea, vomiting along with diarrhea. The most common symptoms reported were fever, cough, shortness of breath, Rhinorrhea, sore throat, and diarrhea. There were no reported deaths in this study. The complete breakdown of percentages for each symptom is given in Appendix Table A1.

#### A Study From Australia

We got the report from the National Incident Room Surveillance Team from Australia of March 7th, 2020 which included all the clinical symptoms, sex, and median age of 71 affected patients (22). It included data on COVID-19 cases diagnosed in Australia, the international situation and the review of the current evidence. The report shows that 14% of confirmed cases were the passengers of the Diamond Princess cruise ship, 23% of patients had a direct or indirect link to the Islamic Republic of Iran and 23% had a direct or indirect link to the mainland China and 21% had no recent travel history. The median age of reported cases was 45 years with a range from 0 to 94 years old. The most common symptoms recorded among the patients were 65% fever, 29% nasal congestion, 35% headache, 71% cough, 50% sore throat, 18% fatigue, 6% nausea, and 26% diarrhea. The report shows the fatality rate of 2.8%.

#### A Study From Canada

Lin et al. (23), reported on March 6th, 2020 the confirmed 135 cases from Ontario Canada. This report included the clinical symptoms, age-range, demographic characteristics, laboratory results, radiographical results, and the median age of affected individuals from January 20th to February 19th, 2020 at 8 hospitals in the Greater Toronto Area. The median age was 28 years. The most common symptoms were: 82% cough, 48% fever, 30% sore throat, 10% diarrhea, and 17% fatigue. There were no deaths reported.

#### A Study From Taiwan

Su and Lai (24), reported the data of 10 confirmed cases till January 31st, 2020, and compared that data to SARS in terms of symptoms, epidemiology, and laboratory characteristics The research shows that there were 30% of males. The most common symptoms included cough in 60%, fever in 50%, flu symptoms in 40%, rhinorrhea in 30%, myalgia in 10%, and shortness of breath in 10% patients. The research concluded that COVID-19 patients are 20 years older than SARS patients and young adults are more susceptible to SARS than elders and children.

#### A Study From Italy

The report from Lombardy Italy had the statistics until March 15th, 2020 stating 22,512 patients, their age range, and sex (25). The report does not include data for the clinical symptoms of the patients. The report also included 2026 cases of COVID-19 among health care workers. The report shows the median age of 64 years with 59.8% of patients being male. The report represents a graph for severity where 24.9% of patients are reported to be severe, 46.1% mild and 5% to be critical patients with 6.7% having few symptoms, 6.7% being asymptomatic, and 10.6 having unspecified symptoms. It also reported 1,625 deaths with a 7.2% fatality rate.

#### A Study From the USA

Arentz et al. (26), reported only about 21 patients of Washington, USA. The report included the clinical symptoms, age-range, median age, and radiological data of all the patients from February 20th, 2020 to March 5th, 2020, at Evergreen Hospital Washington. The report shows 52% of male patients and 86% of the total patients had chronic diseases. The most common symptoms reported in the research shows 52% fever, 76% shortness of breath, and 48% cough. Ninety-five percentage of the patients showed abnormal radiological reports. The report also showed a high rate of ARDS and a high risk of Death. The fatality rate was 52.4%. The detailed data from each of the studies is given in Table A1 in the Appendix of this paper.

## Results

**Figure 2:**
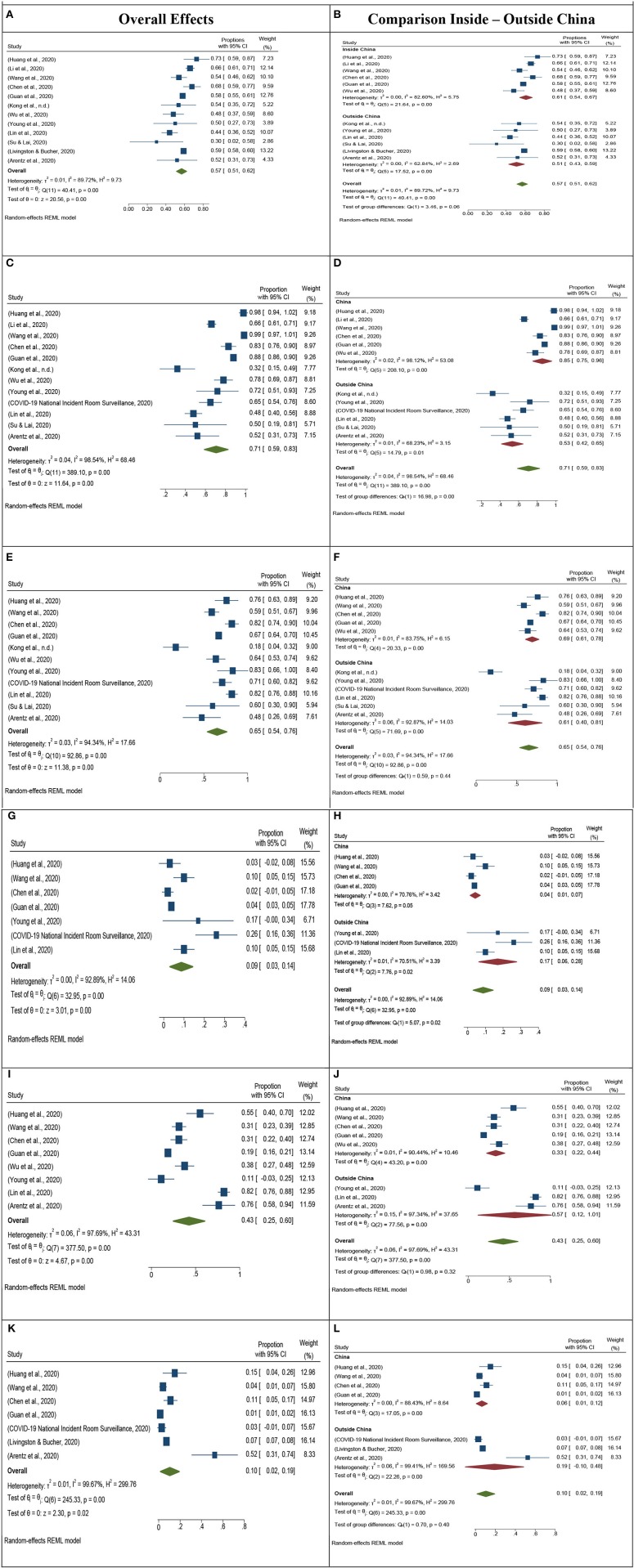
Meta-analysis forest plot overall and comparison of inside and outside of China. **(A)** Forest Plot by using a random-effects model for Gender (Male). **(B)** Comparison of Gender (Male) inside and outside of China. **(C)** Forest Plot by using a random-effects model for fever symptoms. **(D)** Comparison of Fever inside and outside of China. **(E)** Forest Plot by using a random-effect model for cough symptoms. **(F)** Comparison of cough inside and outside of China. **(G)** Forest Plot using Random effect model shown for Diarrhea of 8 studies. **(H)** Comparison of inside and outside of China for Diarrhea. **(I)** Forest Plot using Random effect model shown for 8 studies for Shortness of breath. **(J)** Comparison of inside and outside of China for Shortness of breath. **(K)** Forest Plot using Random Effect model for Fatality rate in 7 studies. **(L)** Comparison of inside and outside of China for a fatality rate.

The meta-analysis of proportions of age, gender, fatality rate, and major clinical symptoms of COVID-19 was done using STATA-16 Software (27), licensed to be used by the corresponding author at the University of Massachusetts Lowell, USA. A random-effect model was used since it was assumed that the symptoms of COVID-19 vary across the populations. The random effect model is a common model that is used to synthesize heterogeneous observations. It is simply the weighted average of the effect sizes of a group of studies which suggests that greater the variability in effect sizes (heterogeneity), the greater the un-weighting until all the studies have equal weight (28). The presence of heterogeneity among the identified studies (Cochran's *Q*) and the extent of heterogeneity (*I*^2^ index) were also examined. Along with the overall heterogeneity in studies, we analyzed heterogeneity in the sub-group for studies conducted in China and outside of China for each of the characteristics. The Forest Plots for all the analyses are listed in Figure 2. The left column shows the overall results of the studies while the right column shows the comparison inside and outside of China.

The median age range is 28–70 in the 13 selected studies. Most of the population lies between the age group 45–60 years. The average median age is 47 ± 7 years. The meta-analysis of gender in the selected studies shows that there are a higher number of male cases compared to female cases (Figure 2A). The null hypothesis that all studies have reported an equal proportion of male cases is rejected. The meta-analysis also shows that there is a significant difference in gender proportions, for studies conducting inside and outside of China (Figure 2B). Moreover, there is a high variation in gender for studies conducted in China as compared to the studies conducted outside of China (Singapore, Taiwan, USA, South Korea, Canada, Australia, and Italy).

The four symptoms of COVID-19 analyzed in this meta-analysis are fever, cough, shortness of breath, and diarrhea (Figure 2). We have found fever to be the most common symptom in the studies, with 71% (CI: 59–83%) cases reporting fever as a symptom of COVID-19 (Figure 2C). Further, we also found that studies conducted inside of China reported a higher percentage of people with fever (61%) as compared to studies conducted outside of China (51%) as shown in Figure 2D. Cough and shortness of breath are also very commonly reported symptoms in studies. The overall reporting of cough symptoms is 65% with 95% Cl is 54–76% (Figure 2E). For shortness of breath, we have observed very different proportions of cases reported inside and outside of China. The overall proportion for shortness of breath inside China is 33% while the proportion for shortness of breath outside China is 57% (Figures 2I,J).

Similarly, we observed contradicting results for the symptoms of diarrhea in COVID-19 cases inside and outside China. The overall proportion of patients reporting Diarrhea as a symptom in China is 4% (CI: 1–7%), while the proportion of patients reporting Diarrhea as a symptom outside China was 17% (CI: 6–28%) (Figures 2G,H).

The fatality rate also shows significant differences in cases reported inside and outside China. The fatality rate for inside China is 6% while, the fatality rate for outside China is 19% (Figures 2K,L). The highest fatality rate outside of China was reported by Arentz et al. (26), in the USA.

## Discussion and Conclusion

The understanding of the epidemiological characteristics of COVID-19 patients is an important research question. The researchers and medical professionals have tried to evaluate the data of patients to identify the most common symptoms which can be used as a yardstick in ruling out the disease while the patient is admitted to a hospital (7). The few most common symptoms which were previously reported in observational studies are fever, cough, sore throat, diarrhea, shortness of breath, and nasal congestion (11). In this study, we have analyzed the patient characteristics including, gender, age, fatality rate, and symptoms of fever, cough, shortness of breath and diarrhea in COVID-19 patients. Our findings suggest that the most commonly reported age group for COVID-19 is 45–60 years. A meta-analysis conducted by Yang et al. (12), suggest that age and comorbidities are highly related in COVID-19 patients. We also found that the male population has a higher proportion in all the studies as shown in Figure 2A, suggesting a higher prevalence of the disease in the male population. The previous meta-analysis has found similar results while studying the gender in COVID-19 (10, 13).

The comparison of studies conducted in China and outside China suggest contrasting and interesting results. Patients in China have a higher proportion of fever, cough, and shortness of breath as compared to patients outside China. However, we found the opposite results for the symptoms of Diarrhea. Patients outside China have a significantly higher proportion of symptoms of Diarrhea as shown in Figure 2H. This can be due to different environmental and social conditions of patients and further investigation can lead to important findings. Our analysis of shortness of breath shows that there wasn't much variation in reporting in all the studies except for Canada and the USA. Both countries have reported the highest shortness of breath. Further investigation can be done on reasons for the high proportion of shortness of breath reported by the patients of COVID-19 outside China. The fatality rate in China is significantly lower as compared to other countries. The highest fatality rate was found in the study conducted in the USA, with a fatality rate of 52% among 21 patients. Our findings also suggest that the fatality rate may increase as the virus spread in countries outside China, which also depends on the health facilities in different counties.

The COVID-19 patients' symptoms, fatality rate, and epidemiological characteristics is an open question for the research community, as more data becomes available, more concrete and stable findings can be uncovered. Also, the clinical symptoms of COVID-19 should not be generalized to fever, shortness of breath and cough only, but other symptoms such as diarrhea are also shown to be prevalent in patients with COVID-19. Our study is the first paper that has conducted a meta-analysis of patient's characteristics comparing observational studies conducted inside and outside China. The findings from this study can help medical and public health professionals as well as the public to better understand the symptoms associated with the COVID-19.

## Data Availability Statement

All datasets presented in this study are included in the article.

## Author Contributions

All authors equally contributed to the study.

## Conflict of Interest

The authors declare that the research was conducted in the absence of any commercial or financial relationships that could be construed as a potential conflict of interest.

## Appendix

**Table A1:** Clinical symptoms.

**References**	**Fever**	**Cough**	**Shortness of breath**	**Sore throat**	**Diarrhea**	**Nasal congestion**	**Headache**	**Sputum production**	**Fatigue/myalgia**	**Nausea or vomiting**	**Other symptoms**
Huang et al. (3)	40 (98%)	31 (76%)	23 (55%)	–	1 (3%)	–	3 (8%)	11 (28%)	18 (44%)		2 (5%)
Li et al. (9)	281 (66%)	–	–	–	–	–	–	–	–	–	238 (56%)
Wang et al. (17)	136 (98%)	82 (82%)	43 (31%)	–	14 (10%)	0(0%)	9 (7%)	37 (27%)	96 (70%)	10 (10%)	53 (39%)
Chen et al. (18)	82 (83%)	81 (82%)	31 (31%)	5 (5%)	2 (2%)	5 (5%)	8 (8%)	–	11 (11%)	11 (1%)	
Guan et al. (2)	975 (88%)	745 (68%)	206 (19%)	153 (14%)	43 (4%)	–	149 (14%)	363 (34%)	418 (38%)	1 (5%)	299 (27%)
Kong et al. (20)	9 (32%)	5 (18%)	–	9 (32%)	–	–	3 (11%)	5 (18%)	4 (14%)	–	5 (18%)
Wu et al. (19)	63 (78 %)	51 (64%)	30 (38%)	–	0 (0%)	5 (6%)	13 (16%)	–	18 (23%)	–	–
Young et al. (21)	13 (72%)	15 (83%)	2 (11%)	11 (61%)	3(17%)	5 (29%)	–	–	–	–	–
COVID-19 National Incident Room Surveillance (22)	46 (65%)	50 (71%)	–	36 (50%)	18 (26%)	–	25 (35%)	–	13 (18%)	4 (6%)	–
Lin et al. (23)	65 (48%)	111 (82%)	111 (82%)	41 (30%)	14 (10%)	41 (30%)	–	–	23 (17%)	–	
Su and Lai (24)	5 (50%)	6 (60%)	–	–	–	–	–	4 (40%)	1 (10%)	–	–
Livingston and Bucher (25)	–	–	–	–	–	–	–	–	–	–	–
Arentz et al. (26)	11 (52%)	10 (48%)	16 (76%)	–	–	–	–	–	–	–	–
